# Research, Clinical, and Sociological Aspects of Autism

**DOI:** 10.3389/fpsyt.2021.481546

**Published:** 2021-04-29

**Authors:** Paul Whiteley, Kevin Carr, Paul Shattock

**Affiliations:** ESPA Research, Unit 133i Business Innovation Centre, The Robert Luff Laboratory, Education & Services for People With Autism Research, Sunderland, United Kingdom

**Keywords:** autism, research, clinical, sociological, knowledge, future

## Abstract

The concept of autism continues to evolve. Not only have the central diagnostic criteria that define autism evolved but understanding of the label and how autism is viewed in research, clinical and sociological terms has also changed. Several key issues have emerged in relation to research, clinical and sociological aspects of autism. Shifts in research focus to encompass the massive heterogeneity covered under the label and appreciation that autism rarely exists in a diagnostic vacuum have brought about new questions and challenges. Diagnostic changes, increasing moves towards early diagnosis and intervention, and a greater appreciation of autism in girls and women and into adulthood and old age have similarly impacted on autism in the clinic. Discussions about autism in socio-political terms have also increased, as exemplified by the rise of ideas such as neurodiversity and an increasingly vocal dialogue with those diagnosed on the autism spectrum. Such changes are to be welcomed, but at the same time bring with them new challenges. Those changes also offer an insight into what might be further to come for the label of autism.

## Introduction

Although there is still debate in some quarters about who first formally defined autism (1), most people accept that Kanner (2) should be credited as offering the first recognised description of the condition in the peer-reviewed scientific literature. The core diagnostic features covering issues in areas of social and communicative interaction alongside the presence of restricted and/or repetitive patterns of behaviour (3) described in his small caseload still remain central parts of the diagnosis today. The core issue of alterations in social cognition affecting emotion recognition and social attention (4) remain integral to the diagnosis of autism. The additional requirement for such behaviours to significantly impact on various areas of day-to-day functioning completes the diagnostic criteria.

From defining a relatively small group of people, the evolution of the diagnostic criteria for autism has gone hand-in-hand with a corresponding increase in the numbers of people being diagnosed. Prevalence figures that referred to 4.5 per 10,000 (5) in the 1960s have been replaced by newer estimates suggesting that 1 in 59 children (16 per 1,000) present with an autism spectrum disorder (ASD) in 2014 (6). The widening of the definition of autism has undoubtedly contributed to the significant increase in the numbers of people being diagnosed. It would be unacceptably speculative however, to define diagnostic changes as being the sole cause of the perceived prevalence increases.

Alongside the growth in numbers of people being diagnosed with autism so there have been changes in other areas related to autism; specifically those related to the research, clinical practice and sociological aspects of autism. Many of the changes have centred on key issues around the acceptance that autism is an extremely heterogeneous condition both in terms of presentation and also in relation to the genetic and biological complexity underlying its existence. That autism rarely exists in some sort of diagnostic vacuum is another part of the changes witnessed over the decades following the description of autism.

In this paper we highlight some of the more widely discussed changes in areas of research, clinical practice and sociological terms in relation to autism. We speculate on how such changes might also further develop the concept of autism in years to come.

### Autism Research

As the definition of autism has subtly changed over the years, so ideas and trends in autism research have waxed and waned. The focus on psychology and behaviour as core descriptive features of autism has, in many respects, guided research and clinical views and opinions about the condition. Social cognition, including areas as diverse as social motivation, emotion recognition, social attention and social learning (4), remains a mainstay of research in this area. The rise of psychoanalysis and related ideas such as attachment theory in the early 20th century for example, played a huge role in the now discredited ideas that maternal bonding or cold parenting were a cause of autism. The seemingly implicit need for psychology to formulate theories has also no doubt played a role in perpetuating all-manner of different grand and unifying reasons on why autism comes about and the core nature of the condition.

As time moved on and science witnessed the rise of psychiatric genetics, where subtle changes to the genetic code were correlated with specific behavioural and psychiatric labels, so autism science also moved in the same direction. Scientific progress allowing the genetic code to be more easily and more cost-effectively read opened up a whole new scientific world in relation to autism and various other labels. It was within this area of genetic science that some particularly important discoveries were made: (a) for the vast majority of people, autism is not a single gene “disorder,” and (b) genetic polymorphisms whilst important, are not the only mechanism that can affect gene expression. Mirroring the role of genetics in other behavioural and psychiatric conditions (7), the picture that is emerging suggests that yes, there are genetic underpinnings to autism, but identifying such label-specific genetic issues is complicated and indeed, wide-ranging.

What such genetic studies also served to prove is that autism is heterogeneous. They complemented the wide-ranging behavioural profiles that are included under the diagnostic heading of autism. Profiles that ranged from those who are profoundly autistic and who require almost constant attention to meet their daily needs, to those who have jobs, families and are able to navigate the world [seemingly] with little or minimal support for much of the time.

It is this heterogeneity that is perhaps at the core of where autism is now from several different perspectives. A heterogeneity that not only relates to the presentation of the core traits of autism but also to how autism rarely manifests in a diagnostic vacuum (8). Several authors have talked about autism as part of a wider clinical picture (9, 10) and how various behavioural/psychiatric/somatic issues seem to follow the diagnosis. Again, such a shift mirrors what is happening in other areas of science, such as the establishment of the Research Domain Criteria (RDoC) project (11). RDoC recognised that defining behavioural and psychiatric conditions on the basis of presented signs and symptoms does not necessarily “reflect” the relevant underlying processes and systems that might be important. It recognised that in order to deliver important clinical information about how and why a condition manifests, or the best strategies to intervene, research cannot just singularly start with the label. Science and clinical practice need more information rather than just a blanket descriptive label such as autism.

To talk about autism as a condition that also manifests various over-represented comorbid labels also asks a fundamental question: is the word “comorbidity” entirely accurate when referring to such labels? (12). Does such comorbidity instead represent something more fundamental to at least some presentations of autism or is it something that should be seen more transiently? Numerous conditions have been detailed to co-occur alongside autism. These include various behavioural and psychiatric diagnoses such as depression, anxiety and attention-deficit hyperactivity disorder (ADHD) (13). Other more somatic based conditions such as epilepsy (14), sleep (15) and various facets of gastrointestinal (GI) functioning (16) have also been discussed in the peer-reviewed science literature. Some of these co-occurring conditions have been described in the context of specific genetic conditions manifesting autism. Issues with the BCKDK (Branched Chain Ketoacid Dehydrogenase Kinase) gene for example, have been discussed in the context of autism, intellectual (learning) disability and epilepsy appearing together (17). Such a diagnostic combination is not unusual; autism often being described as the primary diagnosis with epilepsy and learning disability seen as “add-ons.” But should this be the case? Other evidence pointing to the possibility that epilepsy might under some circumstances beget autism (18) suggests that under some circumstances, such co-occurring conditions are so much more than just co-occurring or comorbid.

Other evidence for questioning the label “comorbid” comes from various animal models of autism. Accepting that one has to be particularly careful about extrapolating from animal models of autism to the more complex presentation of autism in humans (19), various models have suggested that autism may for some, fundamentally coexist with GI or bowel issues (20, 21). Such observations have been noted across different animal models and cover important issues such as gut motility for example, that have been talked about in the context of autism (22).

Similarly, when one talks about the behavioural and psychiatric comorbidity in the context of autism, an analogous question arises about whether comorbidity is the right term. Anxiety and depression represent important research topics in the context of autism. Both issues have long been talked about in the context of autism (1, 13, 23) but only in recent years have their respective “links” to autism been more closely scrutinised.

Depression covers various different types of clinical presentations. Some research has suggested that in the context of autism, depressive illnesses such as bipolar disorder can present atypically (24). Combined with other study (25) suggesting that interventions targeting depressive symptoms might also impact on core autistic features, the possibility that autism and depression or depressive symptoms might be more closely linked than hitherto appreciated arises. Likewise with anxiety in mind, similar conclusions could be drawn from the existing research literature that anxiety may be a more central feature of autism. This on the basis of connections observed between traits of the two conditions (26) alongside shared features such as an intolerance of uncertainty (27) exerting an important effect.

A greater appreciation of the heterogeneity of autism and consideration of the myriad of other conditions that seem to be over-represented alongside autism pose serious problems to autism research. The use of “autism pure” where research participants are only included into studies on the basis of not having epilepsy or not possessing a diagnosis of ADHD or related condition pose a serious problem when it comes to the generalisation of research results to the wider population. Indeed, with the vast heterogeneity that encompasses autism, one has to question how, in the context of the current blanket diagnosis of autism or ASD, one could ever provide any universal answers about autism.

### Autism in the Clinic

As mentioned previously, various subtle shifts in the criteria governing the diagnosis of autism have been witnessed down the years. Such changes have led to increased challenges for clinicians diagnosing autism from several different perspectives. One of the key challenges has come about as a function of the various expansions and contractions of what constitutes autism from a diagnostic point of view. This includes the adoption of autism as a spectrum disorder in more recent diagnostic texts.

The inclusion of Asperger syndrome in the DSM-IV and ICD-10 diagnostic schedules represented an expansion of the diagnostic criteria covering autism. Asperger syndrome defined by Hans Asperger (28) as autistic features without significant language impairment and with intelligence in the typical range, was included in the text for various different reasons. Allen Frances, one of the architects of the DSM-IV schedule, mentioned the importance of having a “*specific category to cover the substantial group of patients who failed to meet the stringent criteria for autistic disorder, but nonetheless had substantial distress or impairment from their stereotyped interests, eccentric behaviors, and interpersonal problems*” (29). It is now widely accepted that the inclusion of Asperger syndrome in diagnostic texts led to an increase in the number of autism diagnoses being given.

More recent revisions to the DSM criteria covering autism—DSM-5—included the removal of Asperger syndrome as a discrete diagnosis on the autism spectrum (30). Instead, a broader categorisation of autism spectrum disorder (ASD) was adopted. The reasons for the removal of Asperger syndrome from DSM-5 are complex. The removal has however generally been positively greeted as a function of on-going debates about whether there are/were important differences between autism and Asperger syndrome to require a distinction (31) alongside more recent revelations about the actions of Asperger during World War II (32). Studies comparing DSM-IV (and its smaller revisions) with DSM-5 have also hinted that the diagnostic differences between the schedules may well-impact on the numbers of people in receipt of a diagnosis (33).

Shifts in the diagnostic text covering autism represent only one challenge to autism in the clinical sense. Other important factors continue to complicate the practice of diagnosing autism. Another important issue is a greater realisation that although the presence of observable autistic features are a necessary requirement for a diagnosis of autism, such features are also apparent in various other clinical labels. Autistic features have been noted in a range of other conditions including schizophrenia (34), personality disorders (35) and eating disorders (36) for examples. Coupled with the increasingly important observation that autism rarely exists in a diagnostic vacuum, the clinical challenges to accurately diagnosing autism multiply as a result.

The additional suggestion of “behavioural profiles” within the autism spectrum adds to the complexity. Terms such as pathological demand avoidance (PDA) coined by Newson and colleagues (37) have started to enter some diagnostic processes, despite not yet being formally recognised in diagnostic texts. Including various autistic traits alongside features such as “resisting and avoiding the ordinary demands of life” and the “active use of various strategies to resist demands *via* social manipulation,” debate continues about the nature of PDA and its diagnostic value (38).

Early diagnosis and intervention for autism have also witnessed some important clinical changes over the years. Driven by an acceptance of the idea that earlier diagnosis means that early intervention can be put in place to “ameliorate” some of the more life-changing effects of autism, there has been a sharp focus on the ways and means of identifying autism early and/or highlighting those most at risk of a diagnosis. It's long been known that there is a heritable aspect to autism, whether in terms of traits or diagnosis (39). In this respect, preferential screening for autism in younger siblings when an older child has been diagnosed is not an uncommon clinical sentiment (40). Other work looking at possible “red flags” for autism, whether in behaviour (41) or in more physiological terms still continue to find popularity in both research and clinical terms.

But still however, autism continues to confound. As of yet, there are only limited reliable red flags to determine or preclude the future presence of autism (42). Early behavioural interventions for autism have not yet fulfilled the promise they are said to hold (43) and autism is not seemingly present in the earliest days of development for all (44, 45). There is still a way to go.

Autism in a modern clinical sense is also witnessing change in several other quarters. The traditional focus of autism on children, particularly boys, is being replaced by a wider acceptance that (a) autism can and does manifest in girls and women, and (b) children with autism age and mature to become adults with autism. Even the psychological mainstay of autism—issues with social cognition—is undergoing discussion and revision.

On the issue of autism presentation in females, several important themes are becoming more evident. Discussions about whether there may be subtle differences in the presentation of autism in females compared to males are being voiced, pertinent to the idea that there may be one or more specific female phenotypes of autism (46). Further characterisation has hinted that sex differences in the core domain of repetitive stereotyped behaviours (47) for example, may be something important when it comes to assessing autism in females.

Allied to the idea of sex differences in autism presentation, is an increasing emphasis on the notion of camouflaging or masking (48). This masking assumes that there may active or adaptive processes on-going that allow females to hide some of their core autistic features and which potentially contributes to the under-identification of autism. Although some authors have talked about the potentially negative aspects of masking in terms of the use of cognitive resources to “maintain the mask,” one could also view such as adaptation in a more positive light relating to the learning of such a strategy as a coping mechanism. Both the themes of possible sex differences in presentation and masking add to the clinical complexity of reliably assessing for autism.

Insofar as the growing interest in the presentation of autism in adulthood, there are various other clinical considerations. Alongside the idea that the presentation of autism in childhood might not be the same as autism in adulthood (49), the increasing number of people receiving a diagnosis in adulthood is a worthy reminder that autism is very much a lifelong condition for many, but not necessarily all (50). The available research literature also highlights how autism in older adults carries some unique issues (51) some of which will require clinical attention.

Insofar as the issue of social cognition and autism, previous sweeping generalisations about a deficit in empathy for example, embodying all autism are also being questioned. Discussions are beginning debating issues such as how empathy is measured and whether such measurements in the context of autism are as accurate as once believed (52). Whether too, the concept of social cognition and all the aspects it encompasses is too generalised in its portrayal of autism, including the notion of the “double empathy problem” (53) where reciprocity and mutual understanding during interaction are not solely down to the person with autism. Rather, they come about because experiences and understanding differ from an autistic and non-autistic point of view. Such discussions are beginning to have a real impact on the way that autism is perceived.

### Autism in Sociological Terms

To talk about autism purely through a research or clinical practice lens does not do justice to the existing peer-reviewed literature in its entirety. Where once autism was the sole domain of medical or academic professionals, so now there is a growing appreciation of autism in socio-political terms too, with numerous voices from the autism spectrum being heard in the scientific literature and beyond.

There are various factors that have contributed to the increased visibility of those diagnosed with autism contributing to the narrative about autism. As mentioned, the fact that children with autism become autistic adults is starting to become more widely appreciated in various circles. The expansion of the diagnostic criteria has also played a strong role too, as the diagnostic boundaries of the autism spectrum were widened to include those with sometimes good vocal communicative abilities. The growth in social media and related communication forms likewise provided a platform for many people to voice their own opinions about what autism means to them and further influence discussions about autism. The idea that autistic people are experts on autism continues to grow (54).

For some people with autism, the existing narrative about autism based on a deficit model (deficits in socio-communicative abilities for example) is seemingly over-emphasised. The existing medical model of autism focusing such deficits as being centred on the person does not offer a completely satisfying explanation for autism and how its features can disable a person. Autism does not solely exist in a sociological as well as diagnostic vacuum. In this context, the rise and rise of the concept of neurodiversity offered an important alternative to the existing viewpoint.

Although still the topic of some discussion, neurodiversity applied to autism is based on several key tenets: (a) all minds are different, and (b) “*neurodiversity is the idea that neurological differences like autism and ADHD are the result of normal, natural variation in the human genome*” (55). The adoption of the social model of disability by neurodiversity proponents moves the emphasis on the person as the epicentre of disability to that where societal structures and functions tend to be “*physically, socially and emotionally inhospitable towards autistic people*” (56). The message is that subtle changes to the social environment could make quite a lot of difference to the disabling features of autism.

Although a popular idea in many quarters, the concept of neurodiversity is not without its critics both from a scientific and sociological point of view (57). Certain key terms often mentioned alongside neurodiversity (e.g., neurotypical) are not well-defined or are incompatible with the existing research literature (58). The idea that societal organisation is a primary cause of the disability experienced by those with the most profound types of autism is also problematic in the context of current scientific knowledge and understanding. Other issues such as the increasing use of self-diagnosis (59) and the seeming under-representation of those with the most profound forms of autism in relation to neurodiversity further complicate the movement and its aims.

The challenges that face the evolving concept of neurodiversity when applied to autism should not however detract from the important effects that it has had and continues to have. Moving away from the idea that autistic people are broken or somehow incomplete as a function of their disability is an important part of the evolution of autism. The idea that autism is something to be researched as stand-alone issue separate from the person is something else that is being slowly being eroded by such a theory.

## Discussion

The concept of autism continues to evolve in relation to research, clinical practice and sociological domains. Such changes offer clues as to the future directions that autism may take and the challenges that lie ahead.

The continuing focus on the huge heterogeneity and comorbidity clusters that define autism are ripe for the introduction of a new taxonomy for describing the condition. A more plural definition—the autisms—could represent one starting position (60) encompassing a greater appreciation that (a) there is variety in the presentation of the core features of autism, (b) there are seemingly several different genetic and biological pathways that bring someone to a diagnosis of autism, (c) different developmental trajectories are an important facet of the autism spectrum, and (d) the various “comorbidities” that variably present alongside autism may offer important clues about the classification of autism. Some authors have stressed that a multi-dimensional conceptualisation may be more appropriate than a categorical concept (61) but further investigations are required.

In relation to the proposed pluralisation of the label, several long held “beliefs” about autism are also ripe for further investigation. The idea that autism is innate and presents in the earliest days in all does not universally hold (45). The finding that some children experience a period of typical development and then regress into autism (62) is becoming more readily discussed in research and clinical circles, albeit not universally so. Similarly, the belief that autism is a lifelong condition for all is also not borne out by the peer-reviewed literature (63). Terms such as optimal outcome (64) might not be wholly appropriate, but do nonetheless, shed light on an important phenomenon noted in at least some cases of autism where diagnostic cut-off points are reached at one point but not another. These and other important areas provide initial support for the adoption of the idea of the plural autisms.

Allied to the notion of “the autisms” is the requirement to overhaul the terminology around the use of the “level of functioning” phrase (65). “High functioning” is typically used to describe those people on the spectrum who present with some degree of communicative language, possess typical or above-average intelligence and who can seemingly traverse the world with only minimal levels of support. “Low functioning”, conversely, is used to describe those with significant support needs who may also be non-communicative. Aside from the societal implications of labelling someone “low functioning” and the possible connotations stemming from such a label, such functioning categorisation do not seemingly offer as accurate a representation as many people might think. The high-functioning autistic child who for example, has been excluded from school on the basis of their behaviour, cannot be readily labelled “high-functioning” if the presentation of their autistic behaviours has led to such a serious outcome. This on the basis that part of the diagnostic decision to diagnose autism is taken by appreciation of whether or not presented behaviours significantly interfere with day-to-day living (3). What might replace functioning labels is still a matter for debate. The use of “levels of support requirement” utilised in current diagnostic criteria offer a template for further discussions. Such discussions may also need to recognise that the traits of autism are not static over a lifetime (51) and support levels may vary as a result.

Whatever terminology is put forward to replace functioning labels, there is a need to address some very apparent differences in the way that parts of the autism spectrum are viewed, represented and included in research. Described as the “understudied populations” by some authors (66) those with limited verbal communicative language and learning disability have long been disadvantaged in research terms and also in more general depictions of autism. In more recent times, there has been a subtle shift to acknowledge the bias that exists against those with a more profound presentation of autism (67). Further developments are however required to ensure that such groups are not excluded; not least also to guarantee the generalisability of autism research to the entire spectrum and not just one portion of it.

On the topic of generalisability to the entire autism spectrum, the moves to further involve those diagnosed with autism in research, clinical and sociological discussions presents opportunities and obstacles in equal measure. The application of the International Classification of Functioning, Disability and Health (ICF) to autism (68) to measure “health-related functioning” represented a key moment in autism participatory research. Taking on board various views and opinions about autism, the development of the ICF core autism sets has allowed those with autism and their significant others to voice their opinions about autism (69).

Such joint initiatives are to be welcomed on the basis of the multiple perspectives they offer including lived experience of autism. But with such participation, so questions are also raised about how representative such opinions are to the entire autism spectrum (70). Questions on whether those who are able to participate in such initiatives “can ever truly speak for the entire autism spectrum?” are bound to follow. Questions also about whether such first-hand reports are more important than parental or caregiver input when it comes to individuals on the autism spectrum are likewise important to ask. This bearing in mind that those with autism participating in such initiatives bring with them the same potential biases as researchers and clinicians carry with them about the nature of autism, albeit not necessarily in total agreement.

The translation of research findings into clinical practice represents another important issue that has yet to be suitably addressed. Although covering a sizeable area, several important stumbling blocks have prohibited the move from “bench to bedside” when it comes to autism research. The focus for example, on the overt behavioural presentation of autism, has in some senses continued to hinder the translational progress of more biological-based findings into autism practice. Nowhere is this seemingly more evident than when it comes to the over-representation of gastrointestinal (GI) issues in relation to autism and their management or treatment. Despite multiple findings of such issues being present (16), very little is seemingly offered despite autism-specific screening and management guidance being in place for nearly a decade at the time of writing (71). Other quite consistently reported research findings in relation to low functional levels of vitamin D (72) for example, have similarly not sparked massive shifts in clinical practices. Ignoring such potentially important clinical features contributes to a state of relative health inequality that is experienced by many on the autism spectrum.

Without trying to prioritise some areas over others, there are some important topics in relation to autism that are becoming important to autism research and clinical practice. Many of these topics are more “real life” focused; taking into account the impact of autism or autistic traits on daily living skills and functioning. These include issues such as the truly shocking early mortality statistics around autism (73) and the need for more detailed inquiry into the factors around such risks such as suicide (74) and self-injury (75) and wandering/elopement (76) alongside the considerable influence of conditions such as epilepsy.

Although already previously hinted at in this paper, the nature of the relationship between autism and various “comorbid” conditions observed to be over-represented alongside is starting to become more widely discussed in scientific circles. Whether for example, moves to intervene to mitigate issues such as depression in relation to autism might also have knock-on effects on the presentation of core autistic features is something being considered. Interest in other topics such as employment, ageing, parenting and the worrying issue of contact with law enforcement or criminal justice systems (77) are also in the ascendancy.

## Conclusions

Autism as a diagnostic label continues to evolve in research, clinical practice and sociological terms. Although the core features described by Kanner and others have weathered such evolution, important shifts in knowledge, views and opinions have influenced many important issues around those core behaviours. As well as increasing understanding of autism, many of the changes, past and present, have brought about challenges too.

## Author Contributions

All authors contributed equally to the writing and review of this manuscript.

## Conflict of Interest

The authors declare that the research was conducted in the absence of any commercial or financial relationships that could be construed as a potential conflict of interest.
